# Phylogeographic diversity of *Orientia tsutsugamushi* strains from clinical isolates in South Korea

**DOI:** 10.1017/S0950268825100988

**Published:** 2026-01-06

**Authors:** Hyungsuk Kang, Yeon-Joo Choi, Changmin Oh, Dong-Min Kim, Yeon-Sook Kim, Kwangjun Lee, Won-Jong Jang

**Affiliations:** 1 Department of Microbiology, School of Medicine, Konkuk University, Republic of Korea; 2 Research Institute of Medical Science, School of Medicine, Konkuk University, Republic of Korea; 3 Institute of Biomedical Science & Technology, Konkuk University, Republic of Korea; 4 Division of Zoonotic and Vector Borne Disease Research, National Institute of Health, Korea Disease Control and Prevention Agency, Republic of Korea; 5 Division of Infectious Diseases, Department of Internal Medicine, Chosun University Hospital, Republic of Korea; 6 Division of Infectious Diseases, Department of Internal Medicine, School of Medicine, Chungnam National University, Republic of Korea

**Keywords:** Orientia tsutsugamushi, Multilocus sequence typing, 56-kDa typing, Vector-borne pathogen, Scrub typhus

## Abstract

*Orientia tsutsugamushi*, the causative agent of scrub typhus, is endemic to the Asia–Pacific region. In South Korea, the Boryong strain is considered dominant; however, nationwide phylogeographic distribution and genetic diversity based on clinical isolates remain incompletely characterized. In this study, 121 *O. tsutsugamushi* clinical isolates were collected from scrub typhus patients at 11 hospitals across South Korea between 2015 and 2024. Isolates were genotyped using *56-kDa* gene sequencing and multilocus sequence typing (MLST) of seven housekeeping genes. Sequence analysis and phylogenetic reconstruction were performed using BLAST, PubMLST, BURST, MEGA11, DnaSP6, and R-based tools. Five *56-kDa* genotypes were identified: Boryong (93.4%), Ikeda, Je-cheon, Young-worl, and Yeo-joo. MLST revealed 11 sequence types (STs), including five novel STs. While the Boryong strain and related STs were distributed nationwide, minor strains showed restricted distribution in northern regions. Several isolates sharing the same *56-kDa* genotype exhibited different MLST STs, indicating possible recombination or local microevolution. This study provides the first nationwide MLST-based characterization of *O. tsutsugamushi* in South Korea and demonstrates the dominance of the Boryong strain alongside localized diversity. Our findings underscore the utility of MLST for higher-resolution typing and support the need for continued molecular surveillance to inform regional epidemiology and disease management.

## Introduction

*Orientia tsutsugamushi* is an obligate intracellular, Gram-negative bacterial pathogen and the agent of scrub typhus [1]. Scrub typhus is a mite-borne zoonotic disease endemic to the Asia–Pacific region [2]. *O. tsutsugamushi* was initially classified under the genus *Rickettsia* and named *Rickettsia tsutsugamushi* [3], but in 1995, it was reclassified into a novel genus, *Orientia*, due to its clearly distinct genetic and phenotypic characteristics [4]. Although *O. tsutsugamushi* was historically restricted to Southern Asia and the Western Pacific, recent studies have reported its presence in Latin America [1] and the Middle East, including a novel species *O. chuto* [1, 5].

The common clinical manifestations of scrub typhus include eschar, rash, fever, headache, and gastrointestinal discomfort [6]. While most infections (~94%) resolve spontaneously, untreated cases can lead to severe complications or even death [7]. In South Korea, the first confirmed case of scrub typhus was reported in 1985 [8], after which the disease became endemic [9]. It is currently designated as a Category III Infectious Disease by Korea Disease Control and Prevention Agency (KDCA), with a reported mortality rate of 0.1–0.3% [10]. KDCA surveillance data from 2001 to the present indicate that the annual number of confirmed cases has remained above 4,000, peaking at 11,105 cases in 2016 [10]. The disease exhibits strong seasonality in Korea, with the majority of cases occurring in November and December [11], and most frequently affects individuals aged 50 to 70 years [12].

Scrub typhus is transmitted by the larval stage (chiggers) of trombiculid mites. In South Korea, the predominant vectors are *Leptotrombidium scutellare* and *L. pallidum*, followed by *L. orientale* and *L. palpale* [13]. These vector species show distinct geographic distributions: *L. pallidum* is more prevalent in the northern regions such as Gyeonggi-do and Gangwon-do, whereas *L. scutellare* dominates in southern provinces like Jeolla-do and Gyeongsang-do [14]. Surveillance and molecular studies have shown that *L. scutellare* primarily harbours *O. tsutsugamushi* strain Boryong and, in rare instances, the Kanda strain. In contrast, *L. pallidum* carries a more diverse set of *O. tsutsugamushi* strains, including Boryong, Je-cheon, and Young-worl [13].

It has been hypothesized that the different *O. tsutsugamushi* strains may be associated with varying clinical manifestations, though this relationship remains unclear [15-17]. In South Korea, scrub typhus has been relatively well studied through multiple surveillance efforts [18, 19], case reports [20, 21], and a co-infection study [22]. The Boryong strain is the most prevalent genotype in Korea [23, 24], and its genome was first sequenced in 2007 [25]. Minor strains such as Young-worl, Je-cheon, Karp, Kawasaki, and Ikeda have also been reported sporadically in mites, rodents, and humans, particularly in the central and northern regions [13, 26].

Most genotyping studies rely on the *56-kDa* type-specific antigen (TSA) gene, which provides discriminatory power [27, 28]. However, to monitor long-term epidemiological trends and evolutionary dynamics, multilocus sequence typing (MLST), which analyses multiple conserved housekeeping genes, has been proposed as a more robust and comparative approach [27, 29].

A recent MLST study using patient isolates collected between 2016 and 2017 in South Korea identified two *56-kDa* genotypes (Boryong, *n* = 49; Karp, *n* = 2) and seven different MLST sequence types (STs), suggesting a relatively conserved *O. tsutsugamushi* population [30]. However, that study was geographically limited to samples from only two tertiary hospitals [30].

Investigating the phylogeographic distribution of *O. tsutsugamushi* strains is essential for understanding the epidemiology of scrub typhus and contributes to broader ecological insights into vector-borne diseases. Therefore, this study aimed to update the phylogeographic landscape of *O. tsutsugamushi* in South Korea. We analysed strain diversity using both *56-kDa* gene typing and MLST, focusing on regional variation across multiple clinical isolates collected between 2015 and 2024.

## Methods

### Isolation of O. tsutsugamushi from scrub typhus patient blood samples

Blood samples were collected from scrub typhus patients at 11 hospitals across South Korea between 2015 and 2024. All samples were screened using nested polymerase chain reaction (PCR), which targeted the *56-kDa* gene with two sets of primers: outer forward (WJ173; 5′-CCAGGATTTAGAGCAGAG-3′) and reverse (WJ174; 5′-CGCTAGGTTTATTAGCAT-3′) primers targeted the 509 bp product size and inner forward (WJ175; 5′-CCTCAGCCTACTATRAKKCC-3′) and reverse (WJ176; 5′-AGCATTTGATAATGCAGCAAGACC-3′) primers targeted the 350 bp product size.

The nested PCR-positive samples were used for *O. tsutsugamushi* isolation. Each sample was centrifuged at 3,000 rpm for 4 min at 4 °C, and 0.8 mL of the buffy coat-containing fraction was inoculated onto Vero cell monolayers in 8-well chamber plates and T25 flasks. Cultures were maintained at 34 °C with 5% carbon dioxide (CO_2_). Successfully obtained clinical isolates were used for further genotyping and their geographical distribution. Genomic deoxyribonucleic acid (DNA) of the isolates was extracted using a commercially available DNA extraction kit (QIAamp DNA Blood Kits, Qiagen, Hilden, Germany) according to the manufacturer’s instructions.

### PCR amplification and sequencing of the 56-kDa type-specific antigen (TSA) gene of the clinical isolates

PCR targeting the *56-kDa* TSA gene for genotyping was performed using a single primer pair, forward (WJ173; 5′-CCAGGATTTAGAGCAGAG-3′) and reverse (WJ794; 5′-CTAGAAGTTATAGCGTACACCTGCACTTGC-3′) primers, which amplified up to a 1,575 bp product size. The PCR cycling condition was initial denaturation at 94 °C for 5 min and 40 cycles of 94 °C for 30 s, 52 °C for 30 s, and 72 °C for 90 s, followed by a final extension at 72 °C for 3 min. The PCR products were visualized on a 1% agarose gel in Tris–acetate–EDTA (TAE) buffer. Positive amplicons were purified using the commercially available kit (FavorPrep GEL/PCR Purification Mini Kit, Favorgen Biotech Corp., Ping Tung, Taiwan), visualized again on another 1% agarose gel in TAE buffer, and subjected to Sanger sequencing for genotyping.

### PCR amplification and sequencing for multilocus sequence typing (MLST) of the clinical isolates

MLST was conducted by amplifying seven housekeeping genes (*gpsA*, *mdh*, *nrdB*, *nuoF*, *ppdK*, *sucB*, and *sucD*) as described in previous studies and according to the *O. tsutsugamushi* PubMLST scheme [29, 31]. Primer sequences, expected amplicon sizes, and sequence lengths used for analysis are shown in Table 1.

**Table 1.** Primer information for *Orientia tsutsugamushi* multilocus sequence typing (MLST) used in this study

Gene	Gene name	Primer	Sequence (5′–> 3′)	Product size^a^	Read size^b^
*gpsA*	Glycerol–3-phosphate dehydrogenase	gpsA_F	TCAGCCCATACTCAAGAAATCA	572	390
		gpsA_R	GCAAATGCCACAATTTCCTT		
*mdh*	Malate dehydrogenase	mdh_F	CCAAAGCAGTTGCTCAAGGT	608	348
		mdh_R	AGCTGCTGCTGGAGCATAAT		
*nrdF*	Ribonucleoside-diphosphate reductase beta subunit	nrdF_F	TAAAGCATGGCACACTCAGC	595	384
		nrdF_R	CTGTTCTGTCCAAACTTCAGGA		
*nuoF*	NADH dehydrogenase chain F	nuoF_F	ATCTGGTTCTATGGCAGTTGAC	645	360
		nuoF_R	CATTTTGCGCCTCTTCTGAGTA		
*ppdK*	Pyruvate, phosphate dikinase precursor	ppdK_F	CAAAGGTGTAACACTTGCTCAGA	591	396
		ppdK_R	TGGTGGTTCATCCATGATTTT		
*sucB*	Dihydrolipoamide S-succinyltransferase	sucB_F	CAGCAAAAGAAAGATGTTCAGC	590	411
		sucB_R	GGTTGCCAAAATGGTAGCAG		
*sucD*	Succinyl-CoA synthase alpha chain	sucD_F	ATGTTCCTCCAGCTTTTGCT	599	411
		sucD_R	TCCAGCGCTTTTTAATGCTT		

*Note:* Primer sequences were adapted from Table 1 in Sonthayanon *et al.* 2010 [29].

a Expected PCR product size.

b Sequence length used for MLST analysis.

The PCR protocol for *gpsA* included an initial denaturation at 94 °C for 4 min, followed by 35 cycles of 94 °C for 30 s, 55 °C for 30 s, and 72 °C for 30 s, with a final extension at 72 °C for 5 min. For all other genes, the annealing temperature was adjusted to 50 °C. PCR products were visualized on a 1% agarose gel in TAE buffer. Positive amplicons were purified using the same purification kit, visualized again on another 1% agarose gel in TAE buffer, and subjected to Sanger sequencing for MLST sequence typing.

### Phylogenetic analysis and geographical distribution

Sequence data for the MLST seven housekeeping genes were aligned individually using MEGA11 (version 11.0.13) [32], and the length was adjusted to the read size in Table 1. The sequencing qualities of both the *56-kDa* gene and MLST genes were verified by corresponding ‘*.ab1’ files. The corrected sequences were analysed for allelic profiles and genetic diversity indices using DnaSP version 6 (V6. 12. 03. x64) [33]. Nucleotide diversity (π) represents the average number of nucleotide differences per site between two sequences. Tajima’s D test was calculated based on the differences between the number of segregating sites and the average number of nucleotide differences. The rates of nonsynonymous (dN) and synonymous (dS) substitutions were also computed, where dN refers to nucleotide mutations that alter the amino acid sequence of a protein, and dS refers to mutations that do not result in amino acid changes. All values were automatically computed using the integrated algorithm of DnaSP V6.

For MLST, each allele sequence was submitted to the *O. tsutsugamushi* PubMLST database [31] to determine allele numbers. Then, the seven housekeeping gene sequences were concatenated into each 2,700 bp contig and submitted to the PubMLST database to obtain MLST STs. When an allele profile did not match any previously defined STs, a new ST was assigned and registered in the database. Clonal complexes using the concatenated contigs were computed by the BURST algorithm implemented on the PubMLST web platform.

The *56-kDa* gene sequences were aligned with reference *O. tsutsugamushi* strains, including strains Boryong (accession: GCF_000063545.1), Ikeda (accession: GCF_000010205.1), Je-cheon (AF430143.1), Young-worl (AF430141.1), and Yeo-joo (AF430144.1). Strain classification was based on phylogenetic clustering using MUSCLE alignment and maximum likelihood tree construction implemented in MEGA11 [34]. Isolates that formed a monophyletic cluster with a reference strain and a pairwise distance of >1% were assigned to that separate strain type.

Geographical distribution maps of *O. tsutsugamushi* strains were generated in the RStudio environment (version 2025.05.0 Build 496) [35], based on hospital locations where the patient blood samples were collected.

## Results

### Descriptive results of clinical isolates and genotyping

A total of 121 *O. tsutsugamushi* were successfully isolated from the collected blood samples of scrub typhus patients at 11 hospitals across South Korea between 2015 and 2024. The number of clinical isolates by year was as follows: 5 clinical isolates in 2015, 16 in 2017, 22 in 2023, and the majority, 78 isolates, in 2024.

Based on *56-kDa* gene sequencing, five distinct *O. tsutsugamushi* strains were identified. The majority of the clinical isolates (*n* = 113) belonged to the Boryong strain, followed by the Ikeda (*n* = 3), Young-worl (*n* = 2), Je-cheon (*n* = 2), and Yeo-joo (*n* = 1) strains.

MLST analysis revealed 11 distinct STs, of which five were newly identified and assigned as ST144, ST155, ST158, ST159, and ST160.

### Genetic diversity of O. tsutsugamushi clinical isolates based on MLST

Genetic diversity was analysed using MLST data derived from the seven housekeeping genes, combining both newly generated sequences and reference data from the *O. tsutsugamushi* PubMLST database (Table 2). As summarized, the number of alleles per gene ranged from 4 to 7, with *sucD* exhibiting the highest allelic diversity. The number of polymorphic sites ranged from 5 to 26, indicating the highest sequence variability in *nuoF.*

**Table 2.** Genetic diversity of *Orientia tsutsugamushi* based on seven housekeeping genes used for multilocus sequence typing

Gene	Length (bp)	No. of alleles	No. of polymorphic sites	GC content (%)	*π* ^a^	*K* ^b^	Tajima’s *D*	dN/dS ratio
*gpsA*	390	5	15	29.6	0.003	1.18	–1.56^c^	0.93
*mdh*	348	4	5	34.8	0.001	0.31	–1.39^c^	0.99
*nrdB*	384	4	6	32.3	0.001	0.54	–1.13^c^	n/a^d^
*nuoF*	360	4	26	37.2	0.007	2.52	–1.47^c^	0.79
*ppdK*	396	5	18	29.5	0.004	1.48	–1.56^c^	0.76
*sucB*	411	4	16	29.6	0.005	1.96	–0.94^c^	0.89
*sucD*	411	7	16	36.6	0.004	1.44	–1.41^c^	0.80
MLST	2,700	11	102	32.7	0.003	9.42	–1.65^c^	0.91

a *π*: Nucleotide diversity [44].

b *K*: Average number of nucleotide differences [45].

c Statistically not significant.

d n/a indicates not applicable due to lack of synonymous sites.

The average number of nucleotide differences (*K*) also varied between loci, with values ranging from 0.31 to 2.52, again highest in *nuoF.* Nucleotide diversity (π) was generally low across all loci, ranging from 0.001 to 0.007.

All Tajima’s D values were negative, suggesting an excess of low-frequency polymorphisms that may indicate recent population expansion or purifying selection. However, none of these values were statistically significant. The dN/dS ratio was below 1 for all loci except *mdh*, supporting the presence of purifying selection across most housekeeping genes.

Table 3 summarizes the clonal complex structure of the 11 MLST STs identified in this study, as determined by BURST analysis.

**Table 3.** Clonal complex analysis of 11 multilocus sequence types identified in this study based on BURST

Clonal complex	MLST STs	Frequency	No. of SLV	No. of DLV	No. of SAT
Clonal complex 1	48	56	2	2	–
	93	51	2	1	1
	94	4	0	1	3
	98	1	1	1	2
	144^a^	1	1	1	2
Clonal complex 2	97	1	1	0	–
	155^a^	1	1	0	–
Clonal complex 3	159^a^	1	1	0	–
	160^a^	1	1	0	–
Singletons	49	2	–	–	–
	158^a^	2	–	–	–

Abbreviations: SLV: single-locus variants; DLV: double-locus variants; SAT: shared allele types.

a Newly identified multilocus sequence types in this study.

Group 1 was the largest complex, comprising five MLST STs (ST48, ST93, ST94, ST98, and ST144), all associated with the Boryong strain based on *56-kDa* genotyping. These STs showed several single-locus variants (SLVs) and double-locus variants (DLVs), indicating a close evolutionary relationship and high intra-group relatedness.

Group 2 consisted of two MLST STs (ST97 and ST155), both linked to the Je-cheon strain. These STs were also SLVs of each other and shared identical alleles across five of the seven housekeeping genes, suggesting recent diversification from a common ancestor.

Group 3 included two novel STs, ST159 and ST160, associated with the Yeo-joo and Ikeda strains, respectively. Although they formed a distinct cluster, their placement outside Groups 1 and 2 indicates limited relatedness to the major clonal complexes identified in this study.

In contrast, ST49 (Ikeda strain) and ST158 (Young-worl strain) did not cluster with any other STs and were therefore classified as singletons. Their exclusion from clonal complexes suggests that they possess unique allele combinations within the dataset, though this does not necessarily imply a higher degree of allelic divergence. Rather, it reflects the absence of closely related types among the analysed isolates.

Newly identified STs in this study are indicated with a superscript ‘a’.

### Comparison of 56-kDa genotypes and MLST STs and phylogenetic relationships

A comparison of the 121 *O. tsutsugamushi* clinical isolates based on *56-kDa* gene sequences and MLST STs is shown in Table 4. The most prevalent genotype was the Boryong strain (*n* = 113), which formed a broader clade in both the MLST and *56-kDa* phylogenetic trees. This strain was further subdivided into five MLST STs: ST48, ST93, ST94, ST98, and the newly identified ST144. Among these, ST48 (*n* = 56) and ST93 (*n* = 51), which share nearly identical allele profiles, were the most common STs identified in this study.

**Table 4.** Comparison of *56-kDa* genotypes, MLST STs, and allele profiles of 121 *Orientia tsutsugamushi* clinical isolates

Strain^a^	MLST ST	Clonal complexes	*n* ^c^	*gpsA*	*mdh*	*nrdB*	*nuoF*	*ppdK*	*sucB*	*sucD*
Boryong	48	Clonal complex 1	56	23	19	19	24	20	19	20
	93		51	23	19	19	24	20	19	38
	94		4	23	19	19	24	34	29	20
	98		1	44	19	19	24	20	119	38
	144^b^		1	23	27	19	24	20	19	20
Je-cheon	97	Clonal complex 2	1	43	27	35	42	39	2	37
	155^b^		1	43	9	35	42	39	2	37
Yeo-joo	159^b^	Clonal complex 3	1	43	27	14	42	39	20	1
Ikeda	160^b^		1	43	27	14	42	39	20	5
	49	Singleton	2	24	20	14	25	21	20	7
Young-worl	158^b^	Singleton	2	50	9	8	18	30	20	32

a Strains determined by *56-kDa* gene sequence (pairwise distances >1%).

b Newly identified MLST STs in this study.

c *n* = number of the clinical isolates assigned to each ST.

In contrast, the remaining strains – Ikeda, Je-cheon, Young-worl, Yeo-joo – were less frequently detected (Table 4) and exhibited greater phylogenetic divergence in both trees (Supplementary Figure S1). Each minor genotype corresponded to one or more unique MLST STs. The Ikeda strain was associated with ST49 and the novel ST160, both of which clustered separately from the Boryong clade, differing at *gpsA*, *nuoF*, and *sucD* loci while sharing several other alleles. The Je-cheon strain included ST97 and novel ST155, which were closely related to the MLST tree and shared identical alleles at five of the seven loci. The Young-worl strain corresponded exclusively to ST158, a novel ST forming an independent lineage with a distinct allele pattern. The Yeo-joo strain was uniquely represented by ST159, another novel type that diverged at multiple loci including *gpsA* and *sucD.*

Overall, five novel STs were identified in this study (ST144, ST155, ST158, ST159, and ST160). These novel STs were mostly associated with minor *56-kDa* genotypes and formed distinct branches in the MLST phylogeny, suggesting localized evolution or recent diversification events.

The tanglegram comparison highlights that while the *56-kDa* gene offers broad strain-level resolution, MLST enables finer differentiation of isolates within the same *56-kDa* genotype. This is particularly evident for the Boryong strain, which appeared homogeneous by *56-kDa* typing but displayed substantial intra-strain heterogeneity when analysed by MLST.

### Geographical distribution of O. tsutsugamushi strains and MLST STs

**Figure 1.**
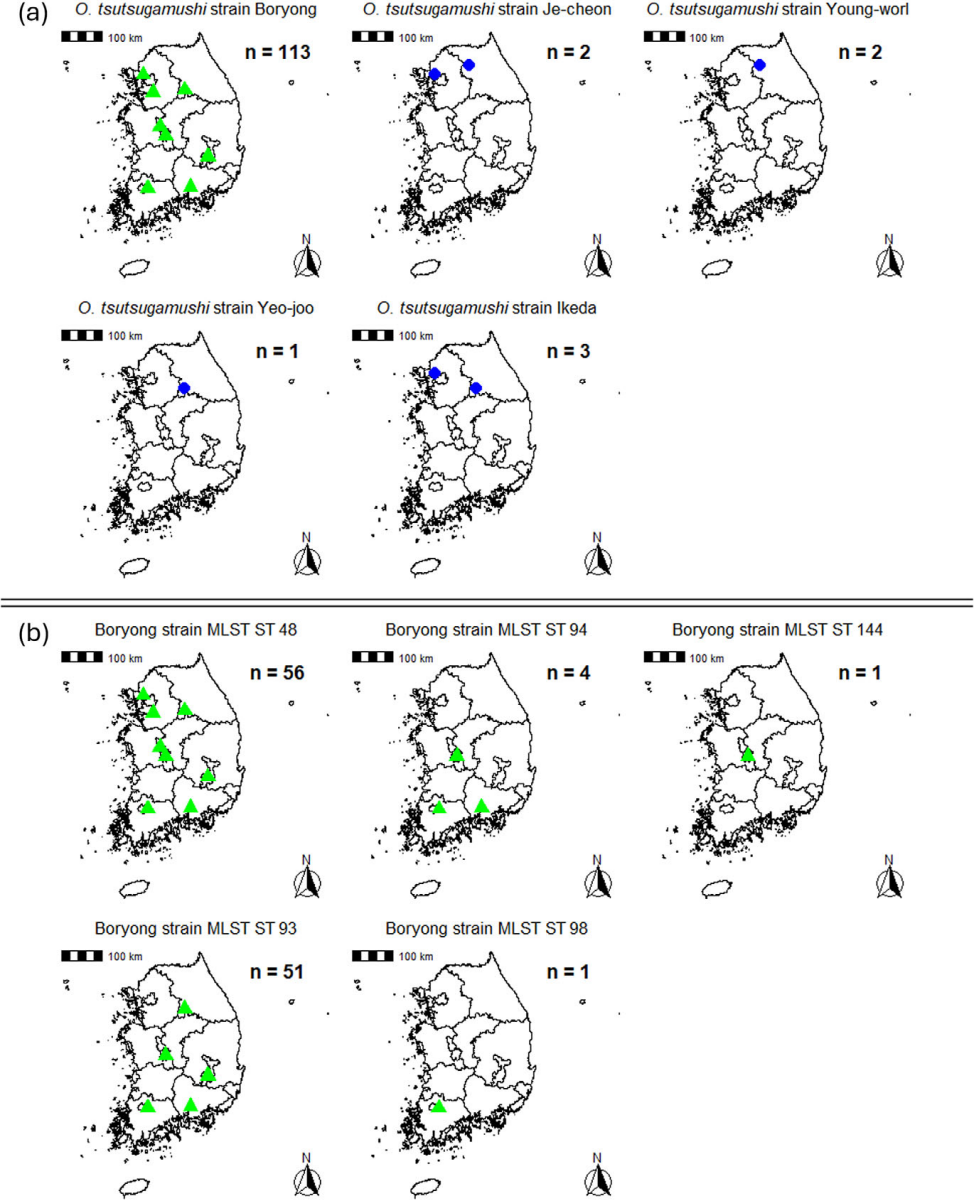
Geographical distribution of O. tsutsugamushi strains in South Korea. (a) Distribution based on 56-kDa gene typing of 121 clinical isolates. (b) Distribution of MLST sequence types (STs) within the Boryong strain (n=113). Each map displays the regional distribution of specific strains or STs. Green triangles indicate hospital where Boryong strain-positive patients were identified, while blue circles indicate hospitals associated with minor strains. All samples were obtained from the blood of scrub typhus patients.

The geographical distributions of *O. tsutsugamushi* clinical isolates, based on *56-kDa* genotyping, and the MLST STs within the major strain, *O. tsutsugamushi* strain Boryong, are shown in Figure 1a,b, respectively. Among the five identified *56-kDa* genotypes, the Boryong strain was the most prevalent (*n* = 113) and was detected across multiple regions throughout South Korea. In contrast, the Ikeda (*n* = 3), Je-cheon (*n* = 2), Young-worl (*n* = 2), and Yeo-joo (*n* = 1) strains were confined to hospitals located in the northern regions. Each of these minor strains was identified in only one or two hospital sites.

Given the predominance of the Boryong strain, we also examined the geographical distribution of its five associated MLST STs (ST48, ST93, ST94, ST98, and ST144). ST48 and ST93 were the most frequently detected and were widely distributed across both northern and southern regions of the country. In contrast, ST94 and ST98 were observed in a limited number of hospitals, and the novel ST144 was detected at a single site.

The presence of multiple MLST STs within the same *56-kDa* genotype, particularly in the Boryong group, indicates that while the strain is widespread, its internal genetic diversity varies regionally. This finding highlights the increased resolution offered by MLST in distinguishing genetic variants within a single antigenic genotype.

## Discussion

In the present study, 121 *O. tsutsugamushi* clinical isolates were successfully obtained from scrub typhus patients across South Korea. Based on *56-kDa* gene sequencing, five distinct genotypes were identified: Boryong, Ikeda, Je-cheon, Yeo-joo, and Young-worl. The Boryong and Ikeda strains were first isolated from patients in 1990 [36] and 1984 [37], respectively. Although the precise dates of initial reports are unclear, the other strains, Je-cheon, Young-worl, and Yeo-joo, had been previously registered in the National Center for Biotechnology Information (NCBI), but no patient-derived isolation had been reported prior to this study. A recent surveillance study of chigger mites in South Korea has also detected these strains, with Boryong being the predominant type (85%), followed by Young-worl (3.8%), Je-cheon (3.4%), and lower frequencies of Yeo-joo (0.4%) and Ikeda (0.4%) [38].

Among the five strains, Boryong was the most prevalent and widely distributed throughout the country. MLST analysis using the seven housekeeping genes revealed intra-strain diversity, with Boryong forming a distinct clonal complex consisting of five STs, including one novel ST (ST144). This finding is consistent with a recent MLST study conducted in a city (Jeonju), South Korea, which identified seven STs, including five novel ones, mostly within the Boryong genotype [30]. In contrast to that study, our analysis additionally found Je-cheon, Yeo-joo, and young-worl strains, further expanding the known strain diversity of clinical isolates in South Korea.

Notably, some minor strains such as Ikeda (ST49) and Young-worl (ST158) did not cluster with other STs, indicating unique allele combinations, though not necessarily higher divergence. This underscores the discriminatory power of MLST over *56-kDa* genotyping.

MLST-based phylogeny revealed that Je-cheon, Yeo-joo, and Young-worl strains, as defined by *56-kDa* typing, clustered more closely with the Japanese Ikeda strain than with Boryong. This suggests possible ancestral relatedness or historical recombination events. The low congruence between MLST and *56-kDa* genotyping is likely due to frequent homologous recombination in *O. tsutsugamushi*, as previously reported [29]. While the *56-kDa* gene is useful for outbreak tracking, it may not reflect broader population structure due to diversifying selection [29].

The use of both *56-kDa* genotyping and MLST STs remains essential, particularly given the ongoing discussion about whether strain types are associated with differences in clinical presentation [24, 39, 40]. Although previous studies have attempted to classify strains as virulent or avirulent, findings have been inconsistent and their relevance to human disease remains unproven [15–17, 40–42]. Thus, further integrated clinical and genomic investigations are required before such associations can be clarified.

Geographically, Boryong was observed nationwide, while the other minor strains were restricted to northern regions. This is consistent with earlier reports from the 1990s, which noted that strains such as Gilliam and Karp were more common in the north, whereas Boryong dominated the central and southern regions [36]. Although ecological factors such as the distribution of mite vectors (*L. pallidum* and *L. scutellare*) and climatic conditions may influence regional patterns [13, 14, 19, 43], our study did not directly investigate these aspects. Future work that integrates vector, rodent, and environmental data with human-derived isolates will be necessary to better understand the ecological drivers of *O. tsutsugamushi* diversity.

In summary, this study provides the first nationwide clinical isolate-based surveillance of *O. tsutsugamushi* in South Korea, confirming the predominance of the Boryong strain and identifying several minor strains with restricted geographical distributions. MLST typing enhanced resolution compared to *56-kDa* gene analysis and uncovered five novel STs. These findings underscore the need for further genomic and clinical investigations to assess potential links between strain diversity and pathogenicity and to track the evolutionary dynamics of scrub typhus in endemic regions.

## Supporting information

10.1017/S0950268825100988.sm001 Kang et al. supplementary material Kang et al. supplementary material

## Data Availability

The datasets used and/or analysed during the current study are available from the corresponding author on reasonable request.

## Supplementary material

The supplementary material for this article can be found at http://doi.org/10.1017/S0950268825100988.
