# Supplementary figures and images for: Phylogeographic diversity of Orientia tsutsugamushi strains from clinical isolates in South Korea
Source: Epidemiol Infect. 2026 Jan 6;154:e14. doi: 10.1017/S0950268825100988 (PMC12835941; doi:10.1017/S0950268825100988)

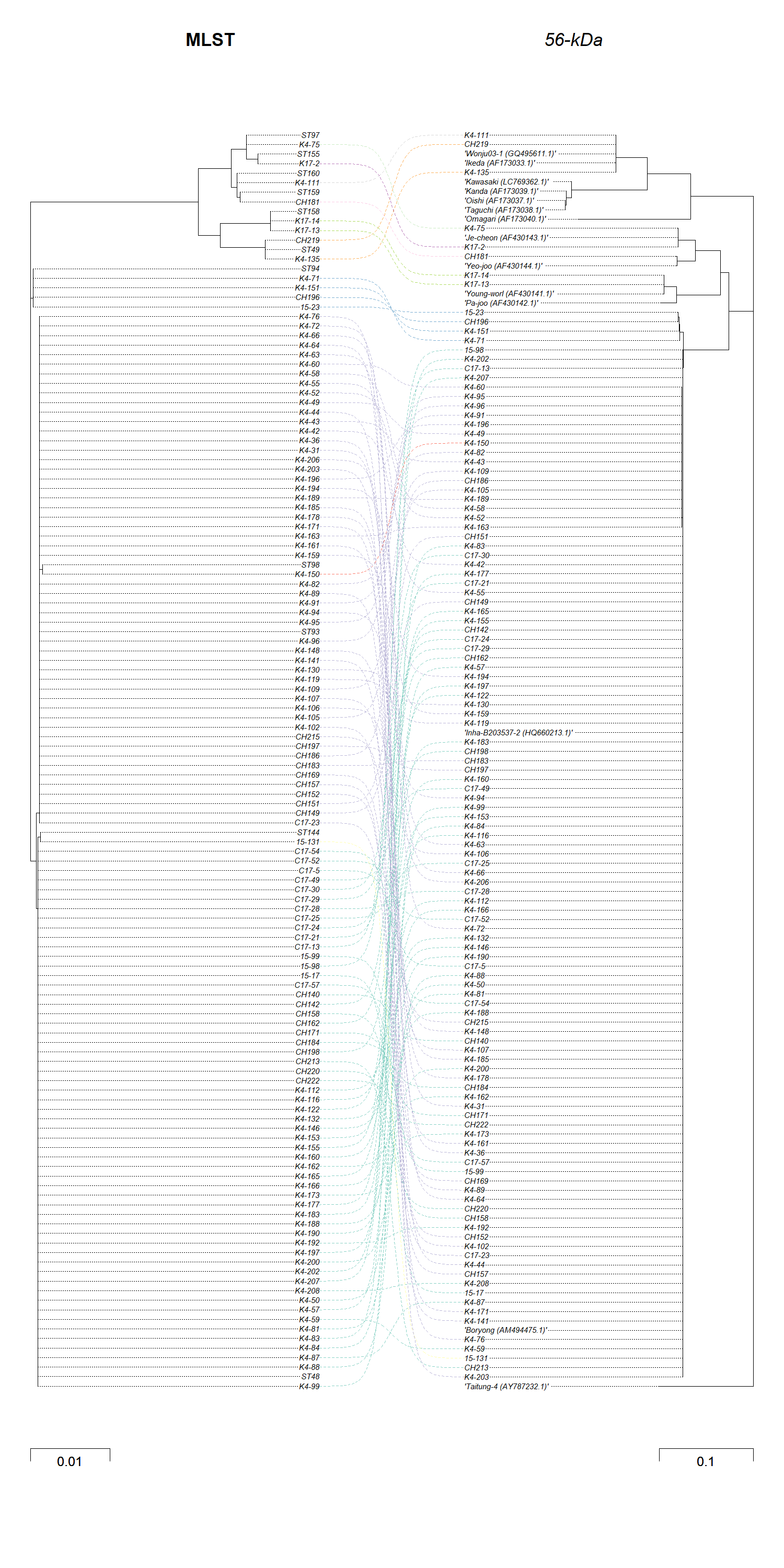

Supplement: Kang et al. supplementary material [file S0950268825100988sup001.tiff]
